# Clinical and molecular characteristics of kinase domain duplications across diverse cancer types in the Chinese population

**DOI:** 10.1002/cam4.5325

**Published:** 2022-11-03

**Authors:** Xiaojing Lai, Ruoying Yu, Qiuxiang Ou, Hua Bao, Xue Wu, Yang Shao, Yang Li, Ying Zhang, Qingqing Ding

**Affiliations:** ^1^ Institute of Cancer Research and Basic Medical Sciences of Chinese Academy of Sciences Cancer Hospital of University of Chinese Academy of Sciences, Zhejiang Cancer Hospital Hangzhou China; ^2^ Geneseeq Research Institute Nanjing Geneseeq Technology Inc. Nanjing Jiangsu China; ^3^ School of Public Health Nanjing Medical University Nanjing Jiangsu China; ^4^ The First Affiliated Hospital of Xi'an Jiaotong University Xi'an Shaanxi P. R. China; ^5^ Department of Pathology Nanjing Jinling Hospital, Nanjing University School of Medicine Nanjing Jiangsu China; ^6^ Department of Geriatric Oncology The First Affiliated Hospital with Nanjing Medical University Nanjing Jiangsu P. R. China

**Keywords:** *EGFR* amplification, kinase domain duplication, targeted sequencing, *TP53* gene alterations

## Abstract

**Background:**

Kinase domain duplications (KDDs) have recently been recognized as oncogenic mutations and possible association with drug resistance in cancers.

**Method:**

Here, targeted sequencing was performed with the tumor tissue and/or plasma from 65 cancer patients with KDDs.

**Result:**

Intact KDDs were identified in approximately 0.1% of the total population across multiple cancer types. *EGFR* KDD was first identified in colorectal cancer and breast cancer, whereas *FGFR2* KDD was first identified in gastric cancer. Tumors with *EGFR* KDD displayed lower concurrent *TP53* gene alterations (*p* = 0.03) and slightly higher chromosome instability (*p* = 0.27) compared to tumors with non‐*EGFR*‐KDDs. Immune pathway analysis further revealed the enrichment of the cytokine receptors pathway (93%) in the KDD carriers. Hyperprogression‐related gene mutations were identified in four cases.

**Conclusion:**

Collectively, our data revealed the genomic features of KDD alterations in a multi‐cancer cohort, providing more information for the potential treatment application in the KDD carriers.

## Graphical abstract

Intact kinase domain duplications were observed at a ratio of 0.1%.The identified KDDs included 55 EGFR KDDs (exons 18–25, exons 17–25), 4 MET KDDs (exons 15–21, exons 12–21), 2 BRAF KDD (exons 10–19, exons 10–18), 1 RET KDD (exons 11–20), 1 FLT3 KDD (exons 6–23), 1 FGFR1 KDD (exons 8–18), and 1 FGFR2 KDD (exons 10–17).

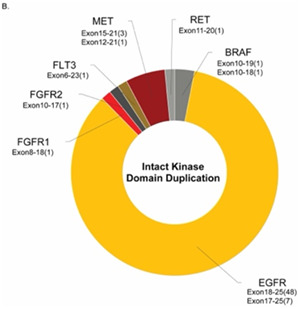

## 1 INTRODUCTION

Kinase domain duplication (KDD) is an intragenic partial duplication that confers cancer cells to the ability to acquire new protein isoforms, resulting in tyrosine kinase activation.^1^, ^2^ So far, oncogenic KDDs are found in *BRAF and EGFR* across different cancer types. *BRAF* KDD has been reported in gliomas and advanced acinic cell tumors, demonstrating response to RAF‐directed therapy.^3^, ^4^ *EGFR* KDD has been identified in non‐small‐cell lung cancer (NSCLC), gliomas, sarcoma, and Wilms'tumor.^5^ Responses to EGFR‐targeted therapies have also been observed among *EGFR* KDD carriers.^6^ Other recurrent KDDs were observed in *MET*, *ROS1*, *ALK*, *KIT*, *FLT3*, *RET*, *FGFR* family genes (*FGFR1‐4)*, *ERBB* family genes *(ERBB2* and *ERBB4)*, *PDGFR* family genes (*PDGFRA* and *PDGFRB*), and *NTRK* family genes (*NTRK*1‐2) from a study involving genomic profiling of 114,200 advanced cancers in a Western population.^7^ However, only the frequencies of the KDDs were revealed in this study.

Furthermore, KDDs have been well recognized as targetable genomic alterations and may be associated with molecular adaptation to selective pressure under cancer therapy, although the underlying resistance mechanism may vary.^8^ For instance, in melanoma, *BRAF* KDD exons 10–18 conferred resistance to BRAF inhibitor treatment.^1^ In one lung cancer patient, the amplification of *EGFR* KDD in the prior treatment sample indicated the involvement of *EGFR* KDD in afatinib resistance.^5^ On the other hand, conflicting evidence was also observed. A case report showed that a lung adenocarcinoma patient harboring *EGFR* KDD demonstrated a response to afatinib.^9^ Moreover, *EGFR* KDD was also implicated in the association with hyperprogression to anti‐PD‐1 immune checkpoint inhibitors in esophageal squamous cell carcinoma.^10^ Another study reported one lung adenocarcinoma patient with *EML4‐ALK* fusion developed *MET*‐KDD as a new resistance mechanism for *ALK* inhibitor ceritinib.^11^ The characterization of KDDs in cancer patients was important, which may affect the disease outcomes. To better understand the genomic features of the KDDs, we retrospectively enrolled 65 KDD‐positive patients with multiple cancer types based on the targeted sequencing results of plasma and/or tumor tissue samples.

## 2 METHODS

A total of 65 patients with intact kinase domain duplication were retrospectively enrolled from a multi‐center database. The written consent was collected from each patient. The next‐generation sequencing (NGS) tests were performed in a centralized clinical testing center (Nanjing Geneseeq Technology Inc.) with a 416‐gene cancer panel and approved by the ethical committee of the participating hospital. The detailed patient sample information was shown in Table S1 and the exclusion and inclusion of patients were shown in Figure S1. The detailed methods were shown in the Supplementary Method S1.

## 3 RESULTS

The distribution of patients with different cancer types is shown in Figure S2A. Lung cancer displayed the highest ratio, accounting for 66% (43,795 out of 66,500) of the total cases. The other frequently observed cancer types included colorectal cancer (10%, 6425 out of 66,500), gastric cancer (4%, 2729 out of 66,500), and breast cancer (3%, 2099 out of 66,500).

Intact kinase domain duplications were observed in 65 cases (0.1%, 65 out of 66,500) with a median age of 54 years old, 85% of which were *EGFR* KDD (Figure S2B, Table S1). There were 37 (57%) females, 26 (40%) males, and 2 (3%) patients with unknown gender information. The identified KDDs included 55 *EGFR* KDDs (exons 18–25, exons 17–25), 4 *MET* KDDs (exons 15–21, exons 12–21), 2 *BRAF* KDD (exons 10–19, exons 10–18), 1 *RET* KDD (exons 11–20), 1 *FLT3* KDD (exons 6–23), 1 *FGFR1* KDD (exons 8–18), and 1 *FGFR2* KDD (exons 10–17). In lung cancer, KDDs were detected at a ratio of 0.1% (56 out of 43,795), including *EGFR* KDD (48), *MET KDD* (4), *BRAF KDD* (2), *FGFR1 KDD*(1), and *FLT3 KDD* (1). The ratio of KDDs is highest in glioblastoma (2%, 3 out of 145) which was all *EGFR‐*KDD. Besides glioblastoma and lung cancer, *EGFR* KDD was also identified in colorectal cancer (3) and breast cancer (1), which has not been reported in previous studies. *MET* KDD was exclusively found in lung cancer, while *FGFR2* KDD was first observed in gastric cancer. *RET* KDD was identified in one case with an unknown cancer type (Figure S2).

**FIGURE 1:**
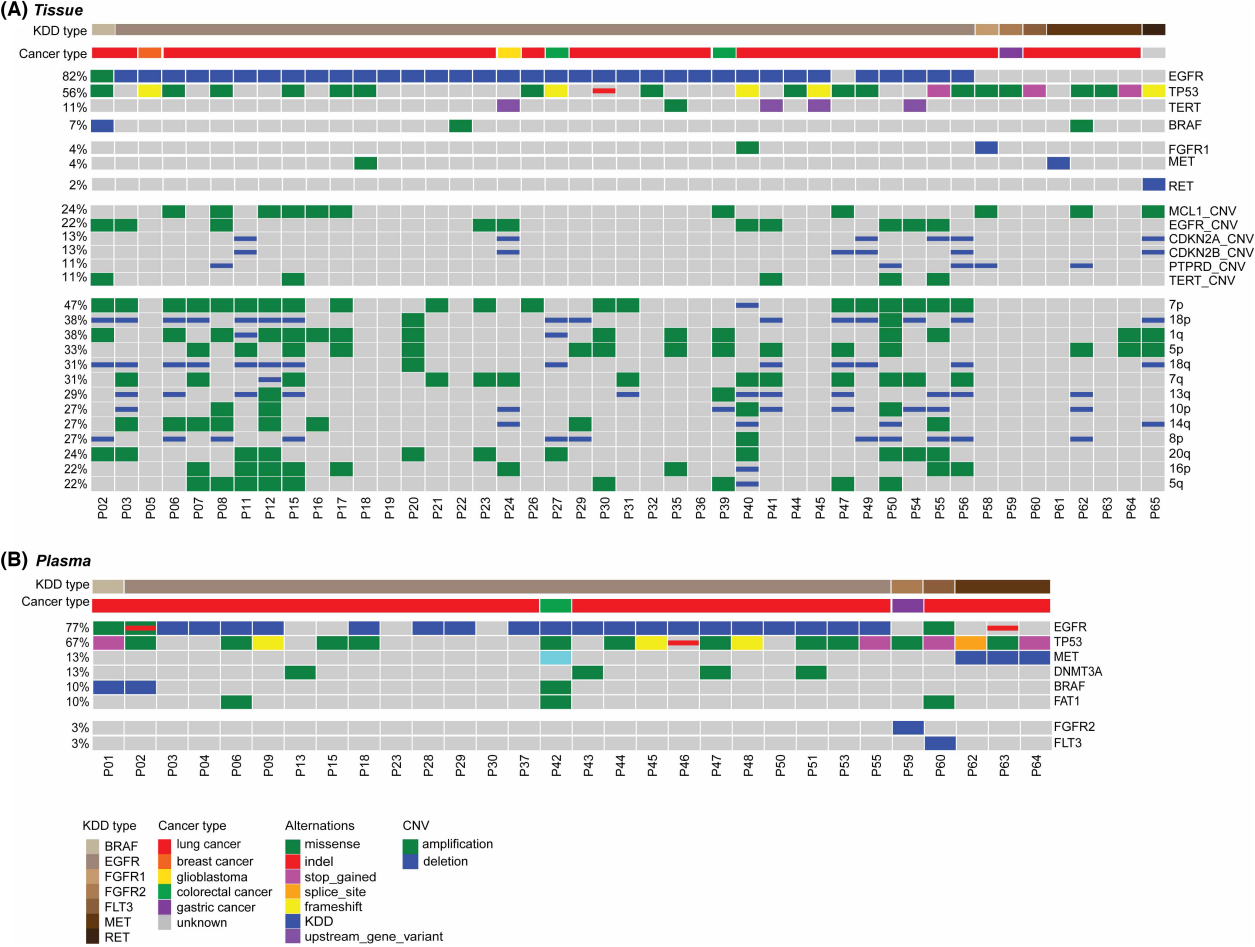
Genomic landscape of patients with kinase domain duplication. (A) Distribution of somatic alterations and copy number variations in tumor tissue from patients with kinase domain duplication (KDD). Each column represents one patient. Cancer type and KDD type were indicated at the top. (B) Distribution of somatic alterations in plasma from patients with KDD. Each column represents one patient. Cancer and KDD types were indicated at the top bar.

The genomic landscape of KDD patients was demonstrated in Figure 1. KDDs were identified in 87% (39 out of 45) of tumor tissue samples (Figure 1A, Table S2) and 87% (26 out of 30) of plasma samples (Figure 1B). In 18 cases with both plasma and tumor tissue, 9 cases had KDD detected in both samples (Figure 1). In tumor tissue, around half of patients with KDD (56%) had concurrent *TP53* alterations including missense, stop gained, and frameshift. The prevalence of concurrent *TP53* alterations (67%) was also observed in the plasma sample (Figure 1B). *MCL* amplification was the most frequently observed copy number variation (CNV), whereas Chromosome 7p CNV was the most frequently observed arm‐level CNV in total KDD carriers. Meanwhile, non‐*EGFR* KDD cases (89%, 8 out of 9) had a higher ratio of *TP53* alterations compared with *EGFR* KDD cases (47%, 17 out of 36) (*p* = 0.03). Compared with non‐*EGFR* KDD, *EGFR* KDD carriers harbored more focal (60% vs 50%, *p* = 0.72) and arm‐level CNVs (82% vs 40%, *p* = 0.01). Twenty‐two percent of KDD cases harbored *EGFR* amplification, 90% (9 out of 10) of which were in *EGFR* KDD carriers (Figure 1A). *EGFR* amplification was not restricted to KDD in lung cancer. A patient with glioblastoma (P24) was identified with *EGFR* KDD and *EGFR* amplification in tumor tissue.

Biomarkers were also assessed in this KDD group. The median chromosome instability score (CIS) of this KDD cohort was 0.32, and the median tumor mutational burden (TMB) was 4.71 muts/Mb. Compared with other genomic characterization studies using similarly targeted panels,^12^, ^13^, ^14^, ^15^ the KDD population had a relatively low TMB, but relatively high CIS. Seven patients had a TMB value higher than 10 muts/Mb, in whom immunotherapy may be considered. The patients with *EGFR* KDD (*n* = 36) displayed a similar CIS (*p* = 0.27) and TMB (*p* = 0.24) compared to the patients with non‐*EGFR* KDDs (*n* = 9), although the median CIS of the *EGFR* KDD group was slightly higher (Figure S3, Table S3).

**FIGURE 2:**
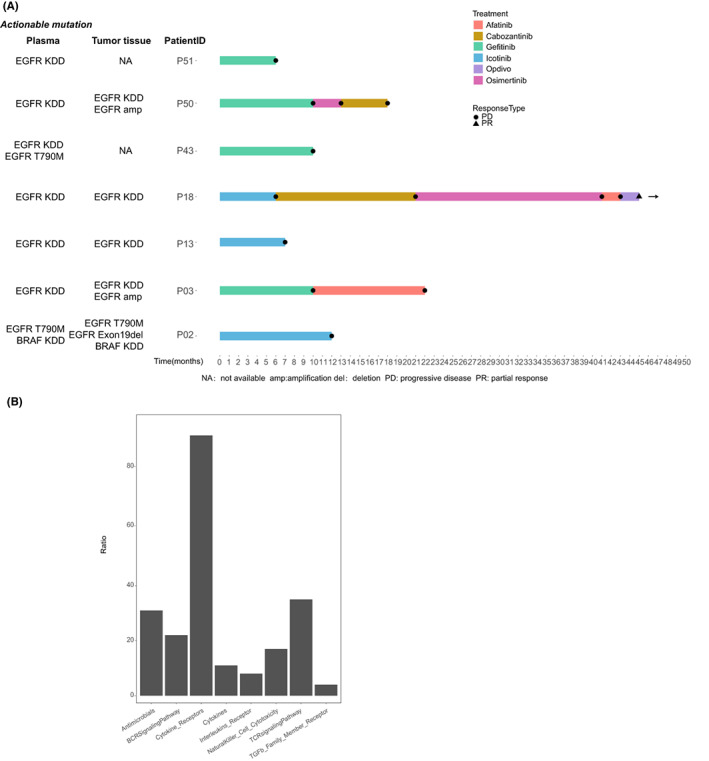
Potential treatment in patients with kinase domain duplication (KDD). (A) Time on treatment. Swimmer's plot of time on treatment for eight patients treated with different tyrosine kinase inhibitors. The actionable mutations included KDD in tumor tissue and plasma were indicated on the left. (B) The ratio of KDD patients with different immune‐related pathways.

Treatment history was available in eight patients with lung cancer and target therapies against *EGFR* and *ERBB2* were administrated (Figure 2A). In P02 and P43, EGFR TKI was administrated and concurrent‐resistant mutation *EGFR* T790M was detected with KDD in tumor tissue or plasma after the patients progressed on the treatment, suggesting KDD was unlikely to be the cause of drug resistance in P02 and P43. In P03 and P50, *EGFR* amplification was found with KDD in afatinib‐treated and erlotinib‐treated tumor tissue samples, respectively. In P13, *EGFR* KDD was detected both in tumor tissue and plasma after the patient progressed on icotinib. Patient 18 underwent several TKIs and *EGFR* KDD was identified in plasma and tumor tissue after afatinib treatment. In P51, *EGFR* KDD was detected in plasma after the patient progressed on gefitinib. No other known resistant mutation was identified in P13, P18, and P51, indicating *EGFR* KDD might associate with drug resistance in these patients.

Recently, *EGFR* KDD has been implicated in association with hyperprogression (HP) during immunotherapy.^10^ A lung cancer patient with *EGFR* KDD has demonstrated considerable efficacy in Nivolumab treatment.^16^ Therefore, we further look into the immune‐related pathway alterations in this KDD cohort. Immune pathway analysis was conducted using the ImmPort database (http://www.immport.org/) and revealed the altered genes in KDD were mostly enriched in the cytokine receptors pathway (93%, Figure 2B). Other frequently altered immune pathways included TCR signaling pathway (35%) and the antimicrobials pathway (31%). Cytokines are the key modulators of immune responses.^17^ Thus, the alterations in the cytokine receptors pathway might associate with the response to the immunotherapy. *STK11/LKB1* mutations and *MDM2/MDM4* amplification have also been implicated in association with HP.^18^ In patients with altered cytokine receptor pathways, *MDM2* amplification was identified in two *EGFR* KDD patients (P11 and P54), whereas *STK11* mutations were identified in one *FGFR1* KDD patient and one *MET* KDD patient (P58 and P62) (Table S2). The administration of immunotherapy in the KDD population could be considered but needs to perform with caution in clinical practice.

## 4 DISCUSSION

Here, we retrospectively studied genomic features and clinical relevancy of intact kinase domain duplication across different cancer types in a large Chinese cohort. KDD was identified at a ratio of 0.097% in this cohort and the majority was in the *EGFR* gene and lung cancer. The high ratio of lung cancer distribution in this Chinese cancer cohort was a major limitation of this study which would have some impact on the interpretation of KDD distribution and frequency. Although KDDs have been reported in the Chinese population,^19^ most of them were confined to lung cancer and a single case study.^6^, ^16^ To the best of our knowledge, our study represented the first study to report the molecular features of KDDs in a Chinese multi‐cancer cohort. Our study may be a good reference for future research carried out on Chinese cancer patients with KDD alterations. Meanwhile, some KDDs were identified in the type of cancer that has not been reported in previous studies. Concurrent mutations were also demonstrated in KDD carriers which may have an impact on the treatment outcome.

*TP53* gene variants are usually found in 35%–60% of NSCLC. It is considered a negative prognostic factor in TKI‐treated NSCLC patients.^20^, ^21^ The association between *EGFR* amplification and better survival from EGFR TKI treatment has also been reported.^2^, ^22^ In this KDD cohort, concurrent *TP53* gene alterations were prevalent with a higher ratio in non‐*EGFR* KDD cases than in *EGFR* KDD carriers. *EGFR* amplification was identified in 22% of KDD cases and was not restricted to lung cancer and *EGFR* KDD. However, due to the lack of treatment history, we cannot evaluate the effect of concurrent *TP53* gene alteration and *EGFR* amplification in KDD carriers. In this cohort, the stage information was available in only 32 patients. Among them, 45% of patients with KDD were stage IV. According to the previous publication, *EGFR* amplification usually correlate with EGFR overexpression and may associate with a more aggressive tumor stage.^23^, ^24^, ^25^ It is highly likely that KDD is associated with late stage given that we observed the enrichment of *EGFR* amplification in this cohort. Another study investigated the prevalence of KDD in Chinese lung cancer patients and showed that 60% of the lung patients with KDD were stage IV.^19^ However, the study did not reveal the genomic features such as concurrent mutations of the KDD carriers. The lack of clinical information also prevented us from further evaluating the association between other molecular and clinical features. Meanwhile, although we observe all the KDDs in the posttreatment samples when patients developed resistance to TKI treatment, we lack the pretreatment samples to compare and further assess the role of KDDs in drug resistance.

Here we also identified the enrichment of the immune‐related pathways especially cytokine receptors pathway in more than 90% of the KDD carriers. The cytokines can be either released in an autocrine fashion to stimulate self‐proliferation, expansion, and drug resistance or in a paracrine fashion to induce recruitment, activation, and differentiation of other cells. The secretion of cytokines into the tumor microenvironment has shown associations with cancer outcomes.^26^ Meanwhile, the released cytokines can bind to cytokine receptors to trigger inflammation and immune response. For instance, the interleukin IL‐17B signals through IL‐17RB receptor to directly promote cancer survival, proliferation, migration, and induces resistance to therapy. High expression of IL‐17B and IL‐17RB has been associated with poor prognosis in different cancer types, including breast cancer, glioblastoma, lung cancer, gastric cancer, and colon cancer. The IL‐17B/IL‐17RB pathway is a signaling cascade that can either directly act on the tumor cells or indirectly leads to tumor microenvironment remodeling.^27^ The alterations in the associated pathways such as MAPK/ERK, PI3K/AKT pathway may also impact the regulation and activation of the signaling cascade.^28^ Chemokine and Chemokine‐receptor signaling is another important cytokine pathway that can reshape the immunological phenotypes within the tumor environment and increase the therapeutic efficacy of immunotherapy. High levels of chemokine receptors in tumor tissues and serum are associated with worse prognosis in multiple cancer types including lung cancer, ovarian cancer, pancreatic cancer, and colorectal cancer.^29^ Further studies are warranted to explore the role of cytokine receptor pathway‐related gene alterations and prognosis in the KDD population.

## 5 CONCLUSIONS

In conclusion, the study interrogated the mutational profiles, molecular biomarkers, and treatment history of cancer patients with KDD alterations and provided insights into optimizing treatment decision‐making for KDD carriers.

## AUTHOR CONTRIBUTIONS

**Xiaojing Lai:** Data curation (lead); formal analysis (lead); writing – original draft (lead). **Ruoying Yu:** Data curation (supporting); formal analysis (lead); methodology (lead); writing – original draft (supporting). **Qiuxiang Ou:** Conceptualization (supporting); methodology (supporting); supervision (supporting); writing – original draft (supporting). **Hua Bao:** Conceptualization (supporting); formal analysis (supporting); methodology (supporting); supervision (supporting); writing – review and editing (supporting). **Xue Wu:** Conceptualization (supporting); methodology (supporting); supervision (supporting); writing – review and editing (supporting). **Yang Shao:** Conceptualization (supporting); methodology (supporting); supervision (supporting); writing – review and editing (supporting). **Yang Li:** Data curation (supporting); formal analysis (supporting); writing – original draft (supporting). **Ying Zhang:** Data curation (supporting); formal analysis (supporting); writing – original draft (supporting). **Qinging Ding:** Conceptualization (lead); supervision (lead); writing – review and editing (lead).

## FUNDING INFORMATION

This work was funded by the 2020 Key Research and Development Program of Shaanxi Province (2020KW‐053).

## CONFLICT OF INTEREST

Ruoying Yu, Qiuxiang Ou, Hua Bao, Xue Wu, and Yang Shao are employees of Nanjing Geneseeq Technology Inc. The remaining authors have no conflict of interest to declare.

## ETHICS APPROVAL AND CONSENT TO PARTICIPATE

The patient consent form was obtained from each patient following the guideline of Institutional Review Board requirements of the First Affiliated Hospital with Nanjing Medical University and the Declaration of Helsinki.

## Supporting information


Appendix S1

Figure S1
Figure S2
Figure S3
Table S1

Table S2
Table S3

## Data Availability

The data sets used and/or analyzed in the current study are available from the corresponding author upon reasonable request.
