# Supplementary material for: Clinical and molecular characteristics of kinase domain duplications across diverse cancer types in the Chinese population
Source: Cancer Med. 2022 Nov 3;12(5):6009–15. doi: 10.1002/cam4.5325 (PMC10028036; doi:10.1002/cam4.5325)
Supplement: Supplementary file 1 — Appendix S1 [file CAM4-12-6009-s001.docx]

**Supplementary Method**

**Patients and sample collection**

A total of 65 patients with intact kinase domain duplication were retrospectively enrolled from a multi-center database from April 2018 to April 2021. In total, 14 cases had only plasma sample and 33 cases were only with tumor tissue samples. Eighteen patients were with both sample types. Two plasma samples were excluded due to failed QC. In patients with available tumor tissue, 45 cases were sequenced with a 416-gene target panel and subjected for further gene alteration analysis. Six tumor samples sequenced with a 139-gene target panel were excluded from genomic landscape analyses because of the difference in the number of genes sequenced. All samples were post-treatment samples.

**DNA library preparation**

Peripheral blood was collected with EDTA-coated tubes (BD Biosciences). Plasma was extracted and shipped to the central testing laboratory for NGS testing. Formalin-fixed paraffin-embedded (FFPE) tumor tissue blocks/sections or fresh tumor tissues were obtained and confirmation for diagnosis and tumor purity was performed by the pathologists.

FFPE were de-paraffinized with xylene and genomic DNA was extracted using the QIAamp DNA FFPE Tissue Kit (Qiagen) following the manufacturer’s protocols. Genomic DNA from fresh tumor tissue and whole blood were extracted using the DNeasy Blood & Tissue Kit (Qiagen) following the manufacturer's protocols. Qualification of purified DNA was performed with Nanodrop2000 (Thermo Fisher Scientific) and quantification of DNA was performed by Qubit 2.0 using the dsDNA HS Assay Kit (Life Technologies) following the manufacturer’s protocols. Sequencing libraries were generated using the KAPA Hyper Prep kit (KAPA Biosystems) and sequenced as previously described[1]. The mean sequencing depths of tumor tissue and plasma were 500X and 3000X respectively.

**Sequencing data processing**

Sequencing data were processed as previously described[1]. The data was demultiplexed and underwent FASTQ file quality control using Trimmomatic to remove low quality data (below 15) or N bases[2]. Qualified reads were mapped to the reference human genome hg19 using Burrows-Wheller Aligner (BWA-mem, v0.7.12; <https://github.com/lh3/bwa/tree/master/bwakit>). Genome Analysis Toolkit (GATK 3.4.0;  <https://software.broadinstitute.org/gatk/>) was used for base quality score recalibration and local realignment around indels. Picard was applied to PCR duplicates remove. VarScan2 was used for the detection of insertion/deletion mutations and single-nucleotide variations (SNVs) [3]. Calls with at least 0.2% mutant allele frequency (MAF) of somatic variant and with at least three supporting-reads from both directions were kept. Common SNVs were excluded with following criteria: 1) present in >1% population in the 1000 Genomes Project or 2) present in the Exome Aggregation Consortium (ExAC) 65,000 exomes database. The resulting mutation list was then filtered with an in-house list of recurrent artifacts from a normal pool of whole blood samples. Sequencing of matched white blood cells from each patient was performed to further eliminate germline variants, sequencing artifacts, clonal hematopoiesis. The Copy number alterations (CNVs) were analyzed by CNVkit and GISTIC algorithm as previously described[4]. FACTERA was applied to structural variants detection using with default parameters[5]. The fusion reads called were manually reviewed and conﬁrmed on Integrative Genomics Viewer (IGV). Tumor mutational burden (TMB) was calculated based on the number of somatic base substitutions and indels in the targeted regions covering 0.85 Mb of coding genome, excluding known driver mutations as they are over-represented in the panel.

**References**

1. Yang Z, Yang N, Ou Q, Xiang Y, Jiang T, Wu X, Bao H, Tong X, Wang X, Shao YW *et al*: **Investigating Novel Resistance Mechanisms to Third-Generation EGFR Tyrosine Kinase Inhibitor Osimertinib in Non-Small Cell Lung Cancer Patients**. *Clinical cancer research : an official journal of the American Association for Cancer Research* 2018, **24**(13):3097-3107.

2. Bolger AM, Lohse M, Usadel B: **Trimmomatic: a flexible trimmer for Illumina sequence data**. *Bioinformatics* 2014, **30**(15):2114-2120.

3. Koboldt DC, Zhang Q, Larson DE, Shen D, McLellan MD, Lin L, Miller CA, Mardis ER, Ding L, Wilson RK: **VarScan 2: somatic mutation and copy number alteration discovery in cancer by exome sequencing**. *Genome research* 2012, **22**(3):568-576.

4. Zhu C, Zhu L, Gu Y, Liu P, Tong X, Wu G, Zhu W, Shen W, Bao H, Ma X *et al*: **Genomic Profiling Reveals the Molecular Landscape of Gastrointestinal Tract Cancers in Chinese Patients**. *Frontiers in genetics* 2021, **12**:608742.

5. Newman AM, Bratman SV, Stehr H, Lee LJ, Liu CL, Diehn M, Alizadeh AA: **FACTERA: a practical method for the discovery of genomic rearrangements at breakpoint resolution**. *Bioinformatics* 2014, **30**(23):3390-3393.
