# Supplementary material for: Clinical and molecular characteristics of kinase domain duplications across diverse cancer types in the Chinese population
Source: Cancer Med. 2022 Nov 3;12(5):6009–15. doi: 10.1002/cam4.5325 (PMC10028036; doi:10.1002/cam4.5325)
Supplement: Supplementary file 2 — Figure S1 Figure S2 Figure S3 Table S1 [file CAM4-12-6009-s002.docx]

**Figure S1. Study Design.**

**Figure S2. The distribution of cancer type and kinase domain duplication type.** (A) Distribution of different cancer type in the overall cohort. The number of cases in each cancer type was indicated in bracket. KDDs detected in each cancer type were in red. (B) Distribution of KDD and duplicated exons in each gene were shown in pie chart. The number of KDD cases was indicated in bracket.

**
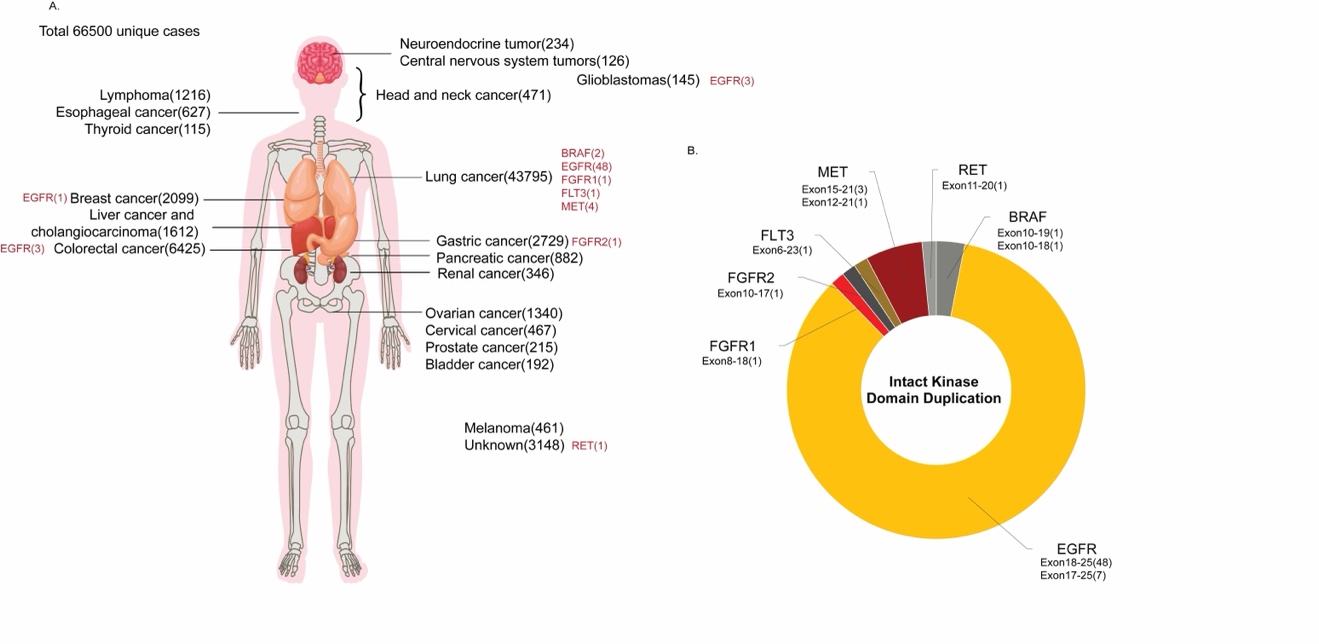
**

**Figure S3. Chromosome instability score and tumor mutational burden of patients with kinase domain duplication.**


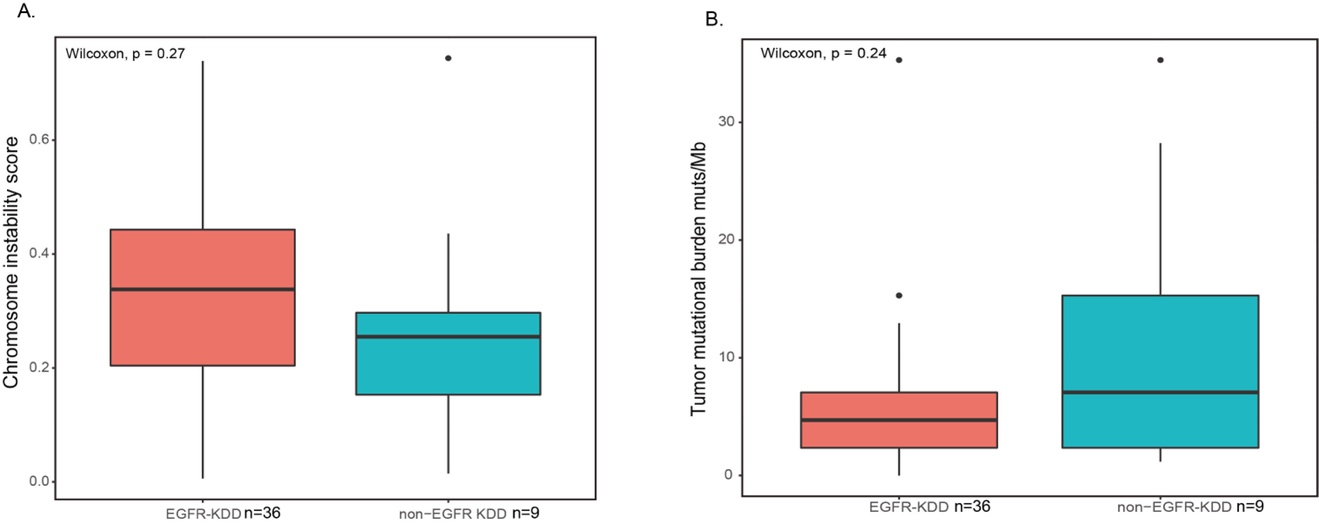


**Table S1. Clinicopathological information of patients with KDD**

| Characteristics | cohort n=65 |
| --- | --- |
| Sex | **No.(Ratio)** |
| Male | 26(40%) |
| Female | 37(57%) |
| Missing information | 2(3%) |
| Age at Diagnose | |
| Range | 23-84 years old |
| Median | 54 years old |
| Missing information | 11 |
| Stage No.(Ratio) | |
| Stage I | 1(2%) |
| Stage III | 2(3%) |
| Stage IV | 29(45%) |
| Missing information | 33(50%) |
| Histology No.(Ratio) | |
| Lung cancer | 56(87%) |
| Glioblastomas | 3(5%) |
| Colorectal cancer | 3(5%) |
| Beast cancer | 1(1%) |
| Gastric cancer | 1(1%) |
| Missing information | 1(1%) |
| KDD type | **No.(Ratio)** |
| EGFR-KDD | 55(85%) |
| MET-KDD | 4(7%) |
| BRAF-KDD | 2(4%) |
| FGFR1-KDD | 1(1%) |
| FGFR2-KDD | 1(1%) |
| FLT3-KDD | 1(1%) |
| RET-KDD | 1(1%) |
